# Does Your Loved One With Cognitive Symptoms Need to See a Doctor? Check It Online

**DOI:** 10.3389/fncom.2022.840200

**Published:** 2022-07-14

**Authors:** Luis Agüera-Ortiz, Manuel Martín-Carrasco, Enrique Arriola-Manchola, Pablo Martínez-Lage, David Andrés Pérez-Martínez, Tomás Ojea, Begoña Soler-López, Guillermo García-Ribas

**Affiliations:** ^1^Psychiatry Department, Instituto de Investigación Hospital 12 de Octubre (i + 12), Madrid, Spain; ^2^Centro de Investigación Biomédica en Red de Salud Mental (CIBERSAM), Madrid, Spain; ^3^Clínica Padre Menni, Pamplona, Spain; ^4^Fundación Matía, Donostia-San Sebastián, Spain; ^5^Area of Neurology, Fundación CITA-Alzheimer Fundazioa, Donostia-San Sebastián, Spain; ^6^Servicio de Neurología, Instituto de Investigación i + 12, Hospital Universitario 12 de Octubre, Madrid, Spain; ^7^Hospital Regional Universitario Carlos Haya, Málaga, Spain; ^8^E-C-BIO, S.L. Departamento Médico, Madrid, Spain; ^9^Servicio de Neurología, Hospital Universitario Ramón y Cajal, IRYCIS, Madrid, Spain

**Keywords:** cognitive decline, cognitive symptoms, memory problems, dementia, online questionnaire, Alzheimer’s disease, IQCODE, AD8

## Abstract

Widespread access to emerging information and communication technologies (ICT) allows its use for the screening of diseases in the general population. At the initiative of the Spanish Confederation of Associations of Families of People with Alzheimer’s disease and other dementias (CEAFA), a website (http://www.problemasmemoria.com) has been created that provides information about Alzheimer’s disease and includes questionnaires to be completed by family or friends concerned about memory problems of a relative. A cross-sectional, randomized, multicenter study was performed to evaluate feasibility, validity, and user satisfaction with an electronic method of completion vs. the current method of paper-based questionnaires for clinically dementia screening completed by the informants: the Informant Questionnaire on Cognitive Decline in the Elderly (IQCODE) and the Alzheimer’s disease-8 screening test (AD8). A total of 111 pairs were recruited by seven memory clinics. Informants completed IQCODE and AD8 questionnaires both in their paper and electronic versions. The correlation between paper and electronic versions was significantly positive for IQCODE (*r* = 0.98; *p* < 0.001) and AD8 (*r* = 0.96; *p* < 0.001). The execution time did not differ significantly, and participants considered their use equally easy. This study shows that an electronic version of the IQCODE and AD8 questionnaires is suitable for its online use via the internet and achieves the same results as the traditional paper versions.

## Introduction

Dealing with the negative consequences of population aging is one of the most important endeavors that health and care-giving systems face, globally. Dementia, particularly Alzheimer’s disease (AD), constitutes a fundamental part of this challenge (WHO, 2012). Among the problems posed by these diseases, procrastinated diagnosis stands out, in particular, leading to delayed management. The existence of effective secondary prevention measures (Luchsinger et al., 2005) and palliative care (Nowrangi et al., 2011) makes the delay in diagnosis even more excruciating. It is estimated that even in developed countries, only 20–50% of patients are correctly diagnosed (Batsch and Mittelman, 2012).

To improve the early detection of AD several strategies have been proposed, with varying degrees of clinical applicability and cost-effectiveness. These include routine screening of the general population, patients seen in primary care or nursing home residents (Woods et al., 2003; Lliffe and Manthorpe, 2004). It should be noted that biomarkers with good properties of sensitivity and specificity and readily applicable to asymptomatic or early symptomatic populations at risk are not yet available (Risacher and Saykin, 2013; Stefani et al., 2013). This leads to an initial disease suspicion still based on cognitive and/or functional complaints that are noticed by patients and/or relatives and considered abnormal enough to seek a consultation (Weir et al., 2011). Most screening techniques are based on the assessment of the affected subject, raising the issue of lack of awareness of illness, often already present in the initial stages of it (Leicht et al., 2010; Degirmenci et al., 2013), and strongly affecting the initiative or willingness to be evaluated. In addition, the practical difficulties of conducting a direct assessment of many potential patients have led to considering distance interviews, either by telephone (Lewis et al., 2001) or the internet (Dougherty et al., 2010; Bateman et al., 2017).

Furthermore, although the role of relatives and/or proxies in the support and care of the patient with AD is fundamental and well established (Schulz and Martire, 2000), their role in the early detection of symptoms as the first step leading to the correct diagnosis is often limited and unrecognized (Cruz et al., 2004). The delay in a correct assessment of the importance of initial symptoms is usually due to incorrect attribution of these symptoms to aging or other clinical entities, such as depression (Werner, 2001). Patients and proxies may usually have doubts as to whether a particular symptom should lead to seeking consultation or not.

Taking all the evidence so far, it seems that a good way to enhance the detection of AD in its early stages would be to make available internet-based screening tests for proxies of potential patients with cognitive impairment.

This is the main motivation for carrying out the AIPAD-online study described below. Its aim is to demonstrate the validity of the online application of a screening test for cognitive impairment, based on the evaluation of an informant with good knowledge of the patient, vs. its traditional paper form.

## Methods

A randomized, multicenter, cross-sectional study was designed to analyze the feasibility, validity, and user satisfaction with the electronic completion method as compared to the usual paper-based standard method.

After approval by the Ethics Committee of the Hospital Regional Universitario Carlos Haya, the study was conducted in the Departments of Neurology, Geriatrics or Psychiatry of seven centers distributed across the Spanish territory. A convenience sample of 100–120 caregivers was estimated, allowing half of the participants starting with electronic completion method and the other half starting with paper-based method of questionnaire completion.

Inclusion criteria comprised subjects older than 50 years who attend as caregivers of outpatients in a specialized memory clinic. The caregiver (informants) must have sufficient knowledge of the patient, usually a first-degree relative or partner living in the same patient’s home, as required by the screening paper versions of the test, and willing to sign an informed consent. Informants having any physical or mental problems were excluded.

The primary objective of the AIPAD-online study was the evaluation of the feasibility of the electronic version of two questionnaires for dementia screening, namely, IQCODE (Informant Questionnaire on Cognitive Decline in the Elderly) short version (Jorm, 1994), and AD8 (Alzheimer’s Disease) (Galvin et al., 2007) and their correlation with the traditional paper version previously translated and validated into Spanish (Morales Gonzalez et al., 1992; Morales et al., 1995; Del-Ser et al., 1997; Carnero Pardo et al., 2013). Both questionnaires were completed by the same informant.

Informants completed both the electronic and paper versions of the questionnaires one at the beginning of the visit and the other at the end. The version order was randomly assigned. A website was developed (see text footnote 1) and sponsored by the Spanish Confederation of Associations of Families of People with Alzheimer’s disease and other Dementias (CEAFA) for the electronic version of the questionnaires, and previously validated paper version were used (Morales Gonzalez et al., 1992; Morales et al., 1995; Del-Ser et al., 1997; Carnero Pardo et al., 2013). The sentence formulation of the items was identical in both versions.

The IQCODE questionnaire is a tool for the detection of cognitive impairment and dementia in older people that is completed by a caregiver or family member with a relationship with the patient for at least five previous years. The short version of the IQCODE can be completed in 10–15 min with almost no influence of education (Jorm, 1994). The questions refer to the situation of the elderly person compared to the one they presented 5 or 10 years ago. Each question is answered with a five-point Likert-type scale with scores ranging from 1 to 5: Much improvement = 1 point; Little improved = 2 points; It has hardly changed = 3 points; It has gotten a little worse = 4 points; It has gotten very bad = 5 points. The total score is calculated by the sum of the scores divided by 17, so the final score range is 1–5 points. A higher score means greater cognitive decline. Cronbach’s alpha coefficient has been calculated in seven studies, with a range of 0.93–0.97. The total score can also be calculated with the sum of the scores for each question, with a range of 17–85 points (Jorm, 1994).

The AD8 questionnaire is a very brief informant questionnaire containing just 8 yes/no questions. Its diagnostic accuracy for both cognitive decline, dementia, and AD has been subjected to rigorous validation. The total score of the AD8 is equal to the number of affirmative answers (Galvin et al., 2007).

Demographic variables of the patients and informants, questionnaire results in both versions, the time for completion of the questionnaires in both systems, and a questionnaire for satisfaction and usability of both versions were collected in an ease-of-use Likert type scale ranging from 1 (not easy at all) to 5 (very easy). To obtain a description of the sample, in subsequent visits, clinical diagnostic impression was collected, based on the NINCDS-ADRDA criteria for probable AD as patients with Alzheimer’s disease was the only diagnosis observed (Tierney et al., 1988), or Petersen criteria for mild cognitive impairment (MCI) (Petersen et al., 1999). No other dementia severity assessment was recorded for the study.

Statistical analysis included descriptive quantitative and qualitative variables of the sample and Spearman’s correlation between the IQCODE and AD8 questionnaires in their paper vs. electronic versions. The SPSS 14.0 statistical analysis program (Chicago, IL.) for the study of the data was used.

## Results

A sample of 118 cases in electronic format and 113 cases in paper format was obtained. Seven questionnaires/patients did not meet the inclusion criteria, and consequently, the final sample consisted of 111 cases for which information was available both electronically and in paper format. A total of 73 patients (65.8%) and 75 of informants (65.6%) were women. Mean age was 77.8 years old (range, 60–97) for patients and 57.4 years old (range, 32–92) for informants.

The most frequent educational levels of patients were basic education (ISCED levels 1–2 (International Standard Classification of Education, ISCED 2011, UNESCO) in 66 patients (59.5%), Upper secondary education (ISCED level 3) in 13 patients (11.7%), and university education (ISCED levels 4–8) in 21 patients (18.9%). Eleven patients (9.9%) did not have education level. For the informants, the percentages were 29 (26.1%) with basic education, 39 (35.1%) with upper secondary education, and 42 (37.8%) with university education, with only one informant (0.9%) without education level. The type of relationship of patients with the informants was most commonly a sibling in 57 (51.8%) and partner in 30 (27.3%). Most informants, 79 (71.2%), saw the patient daily, 19 (17.1%) saw the patient every 2–3 days, and 12 (10.8%) saw the patient once a week. In one case (0.9%), the patient was visited once a month. Reasons for the consultation were memory loss in 66 patients (60%), behavioral disorder in 10 patients (9.1%), and cognitive impairment not otherwise specified in 14 patients (12.7%). Combined consultation reason was observed in 21 patients (21%). Data were not detailed in one patient.

A total of 57 patients (51.3%) included met the NINCDS-ADRDA criteria for probable AD and 42 (39.3%) of them met the criteria for MCI. Four patients were not classified for either MCI or probable AD.

Total scores of the IQCODE and AD8 questionnaires are displayed in Table 1. In the case of the total scores calculated as a sum of the responses, the scores were converted to a percentage scale to make them more readily interpretable. No significant statistical differences were observed in the mean scores between electronic and paper questionnaire versions.

**TABLE 1:** Summary statistics of the total scores of the IQCODE and AD8 questionnaires, ease of use, completion times, and reliability.

		Mean (SD)*
Mean score (range of scores, from 1 = he/she has improved much to 5 = he/she has become much worse)	Electronic IQCODE paper IQCODE	4.08 (0.65) 4.05 (0.64)
Total score IQCODE (maximum 100)	Electronic IQCODE paper IQCODE	71.39 (20.39) 70.24 (21.56)
Total score AD8 (maximum 8)	Electronic AD8 paper AD8	5.66 (2.36) 5.70 (2.21)
Ease of use (range from 1 = not easy at all to 5 = very easy)	Electronic IQCODE paper IQCODE	3.88 (0.92) 3.95 (0.84)
	Electronic AD8 paper AD8	4.06 (0.79) 4.07 (0.73)
Completion time for electronic IQCODE Completion time for paper IQCODE		03:32 Min (01:38) 03:08 Min (01:08)
Completion time for electronic AD8 Completion time for paper AD8		01:41 Min (00:50) 01:44 Min (00:56)
Reliability of the electronic and paper versions of the IQCODE and AD8 questionnaires	Cronbach’s Alpha	
	Electronic IQCODE paper IQCODE	0.95 0.96
	Electronic AD8 paper AD8	0.79 0.75

*P > 0.05 for all the electronic vs. paper versions comparisons.

User satisfaction was very similar for electronic and paper versions (Table 1). Spearman’s correlation coefficient was calculated to analyze the degree of association between easiness of completion of the electronic and paper versions of the IQCODE (*r* = 0.84) and AD8 (*r* = 0.88) questionnaires. Correlations were high, positive, and statistically significant (*p* < 0.01). Consistent with this high degree of association between the electronic and paper versions, there were not statistically differences between the ease of completion of the two versions of the IQCODE and AD8 questionnaires. Completion times of the scales were similar, although slightly higher in the case of electronic versions (Table 1). Also, internal consistency and reliability analysis were high for both tests in paper and electronic versions (Table 1).

The analysis of the correlation in the two versions of the tests, convergent validity, was very high as shown in Table 2.

**TABLE 2:** Spearman’s correlation between the versions of the IQCODE and AD8 questionnaires.

	Paper AD8 total score	Paper IQCODE sum score	Paper IQCODE total score
Electronic AD8 total score	0.96 (*)		
Electronic IQCODE sum score		0.98 (*)	
Electronic IQCODE total score			0.98 (*)

*The correlation is significant at two-tailed p-value of 0.01.

## Discussion

This is the first study to compare an informant-based method of cognitive impairment screening on paper with its online version, showing no significant statistical differences between both administration methods of screening for AD by traditional methods—usually questionnaires on paper that are self-completed or completed by an informant—have shown good predictive achievement (Lischka et al., 2012). The performance of similar procedures through a website involves uncertainties related to the ecological environment of the application of the test or questionnaire that raise questions that this study aims to answer. These questions primarily involve the fact of whether there are any differences when answering to the questionnaire through the computer media compared to the traditional method by people with varying degrees of familiarity with the use of computers, especially informants of a certain age.

In addition, the design of the website containing the evaluation procedure should have specific characteristics of simplicity, ease of use, and minimization of use options to reduce variability. The creation of the website www.problemasmemoria.com containing the assessment questionnaires entailed a series of discussions by experts and reviewing various versions until arriving at the final version, which is the one that was tested and that appears on the website above. Its content includes basic data in relation to both the person being evaluated and the evaluator, and additional information of a clinical nature concerning the individual being evaluated. The fundamental core of the website includes the assessment of the potential patient. This is done through the versions validated in Spain of the two questionnaires that are most widely used for the detection of dementia based on the data given by an informant: the IQCODE and AD8 questionnaires (Hendry et al., 2019; Burton et al., 2021; Quinn et al., 2021). The inclusion of both instruments was designed to compare the performance of both questionnaires and possibly to decide to use only one in the case of developing a shortened version for the website.

Questionnaires were selected based on the evaluation of the informant to avoid the tendency of patients with cognitive impairment to minimize their deficits and therefore unconsciously distort the results and also because, despite its convenience, these potential patients may not want to cooperate in assessing their own cognitive or functional abilities.

The sample taken for the comparative study of the online and paper versions of the two assessment instruments does not differ from the population that regularly came for specialized consultation for memory or cognitive complaints from the socio-demographic point of view and neither in relation to caregivers. It should be noted that the highest proportion of caregivers was made up by children of the person being evaluated, who had a nearly daily relationship with the person.

The results of the paper and online versions of the two questionnaires were virtually identical. The reliability and convergent validity were highly significant, with Cronbach’s alpha values in the upper range. In addition, both ease of use and satisfaction of the informants was similar for both the paper as well as the electronic versions, which provides strong support for the electronic application. Both versions were completed in a similar amount of time, though marginally longer in the electronic version, probably related to the lower familiarity with operating a computer vs. the use of paper. In both cases, it involved a reasonably short time.

This experience of dementia screening supported by a website available online is the first to use the information from an informant. There are other experiences, but they are based on information provided by the subject being evaluated, primarily based on the performance of cognitive tests moved to the internet. Thus, Dougherty et al. (2010) used a new battery of multi-domain cognitive tests with a period of application lasting more than 15 min. Therefore, this requires a good level of cooperation from the subject being evaluated. Brandt and Rogerson (2011) also use for this purpose an episodic memory test that is not yet validated. This involves a time for encoding information so requires the subject to be evaluated as a collaborator. Wesnes et al. (2017) have reported positive preliminary data using a new cognitive battery of four tests validated in their paper version, but not online. The results of these experiments are only partially comparable to that presented here, as they involve direct evaluations of the subject rather than information gathered by a reliable informant, although all of them reinforce the idea that this type of screening is feasible and has acceptable predictive capabilities.

Our study has limitations such as the number of participants and the selection bias in relation to a sample recruited in the medical setting. Also, the study was limited to patients with cognitive symptoms, so all questionnaires had high values. We did not consider including a control group of volunteers with no cognitive complaints as we expected that the visits to the webpage mainly will be of people worried about initial cognitive symptoms. Although 10.8% of the informants saw the patient every week, and this could derive in lower knowledge about the patient mental status, all the informants fulfilled the requirement of the validated questionnaires, and the way they are usually applied. However, in our opinion, these limitations do not invalidate the primary objective of the study: To evaluate the possible differences between traditionally presented tests vs. a test conducted in an online platform. A limitation to transfer these results to the general population is the difficulty to access internet in some socioeconomic levels. There is a cultural constraint for some population groups that has been called digital illiterates. For this reason, our study evaluates tests designed for caregivers and relatives of patients who often have younger age and more access to the internet. However, we believe that limited access to the internet will become less important in the future, even for elderly populations. Although the work was performed long before the COVID-19 pandemics, it raises more importance of the availability of web-based questionnaires minimizing in-office consultations. The electronic version of the questionnaire proposed in this work might add a new useful tool for the becoming years as this pandemic, or others to come, will change our interpersonal and patient-doctor relationships.

In conclusion, the electronic versions of the IQCODE and AD8 questionnaires presented on the website www.problemasmemoria.com constitute a valid and reliable method, comparable to the paper versions for dementia and cognitive impairment detection, with high rates of acceptability by informants evaluating the subjects, who perform this activity in a reasonably short time. These results warrant further studies to validate the diagnostic performance of the electronic versions administered online and their contribution to reducing the time to diagnosis and improving early detection of AD and other dementias.

## Data Availability Statement

The raw data supporting the conclusions of this article will be made available by the authors, without undue reservation.

## Ethics Statement

The studies involving human participants were reviewed and approved by the Hospital Regional Universitario Carlos Haya. The patients/participants provided their written informed consent to participate in this study.

## Author Contributions

GG-R drafted the manuscript. All authors contributed to the conceptualization, methodology design, review of the analysis of the study data, contributed to the final form, and approved the manuscript.

## Conflict of Interest

The authors declare that the research was conducted in the absence of any commercial or financial relationships that could be construed as a potential conflict of interest.

## Publisher’s Note

All claims expressed in this article are solely those of the authors and do not necessarily represent those of their affiliated organizations, or those of the publisher, the editors and the reviewers. Any product that may be evaluated in this article, or claim that may be made by its manufacturer, is not guaranteed or endorsed by the publisher.

## References

[B1] Bateman D. R. Srinivas B. Emmett T. W. Schleyer T. K. Holden R. J. Hendrie H. C. (2017). Categorizing health outcomes and efficacy of mhealth apps for persons with cognitive impairment: a systematic review. *J. Med. Internet Res.* 19:e301. 10.2196/jmir.7814 28855146 PMC5597798

[B2] Batsch N. L. Mittelman M. S. (2012). *World Alzheimer Report 2012. Overcoming the Stigma of Dementia.* London: Alzheimer’s Disease International.

[B3] Brandt J. Rogerson M. (2011). Preliminary findings from an internet-based dementia risk assessment. *Alzheimers Dement.* 7 e94–e100. 10.1016/j.jalz.2010.08.229 21220214

[B4] Burton J. K. Fearon P. Noel-Storr A. H. McShane R. Stott D. J. Quinn T. J. (2021). Informant Questionnaire on Cognitive Decline in the Elderly (IQCODE) for the detection of dementia within a secondary care setting. *Cochrane Database Syst. Rev.* 7:Cd010772. 10.1002/14651858.CD010772.pub3 34278561 PMC8406705

[B5] Carnero Pardo C. de la Vega Cotarelo R. López Alcalde S. Martos Aparicio C. Vílchez Carrillo R. Mora Gavilán E. (2013). Assessing the diagnostic accuracy [DA] of the Spanish version of the informant-based AD8 questionnaire. *Neurologia* 12 538–546. 10.1016/j.nrl.2012.03.012 22652137 PMC3485452

[B6] Cruz V. T. Pais J. Teixeira A. Nunes B. (2004). The initial symptoms of Alzheimer disease: caregiver perception. *Acta Med. Port* 17 435–444. 16197855

[B7] Degirmenci E. Degirmenci T. Duguncu Y. Yilmaz G. (2013). Cognitive insight in Alzheimer’s disease. *Am. J. Alzheimers Dis. Other Dement.* 28 263–268. 10.1177/1533317513481089 23493721 PMC10852787

[B8] Del-Ser T. Morales J. M. Barquero M. S. Cantón R. Bermejo F. (1997). Application of a Spanish version of the “Informant Questionnaire on Cognitive Decline in the Elderly” in the clinical assessment of dementia. *Alzheimer Dis. Assoc. Disord.* 11 3–8. 10.1097/00002093-199703000-00002 9071438

[B9] Dougherty J. H. Jr. Cannon R. L. Nicholas C. R. Hall L. Hare F. Carr E. (2010). The computerized self-test [CST]: an interactive, internet accessible cognitive screening test for dementia. *J. Alzheimers Dis.* 20 185–195. 20164591 10.3233/JAD-2010-1354

[B10] Galvin J. E. Roe C. M. Coats M. A. Morris J. C. (2007). Patient’s rating of cognitive ability. using the AD8, a brief informant interview, as a self-rating tool to detect dementia. *Arch. Neurol.* 64 725–730. 10.1001/archneur.64.5.725 17502472

[B11] Hendry K. Green C. McShane R. Noel-Storr A. H. Stott D. J. Anwer S. (2019). AD-8 for detection of dementia across a variety of healthcare settings. *Cochrane Database Syst. Rev.* 3:Cd011121. 10.1002/14651858.CD011121.pub2 30828783 PMC6398085

[B12] Jorm A. F. (1994). A short form of the Informant Questionnaire on Cognitive Decline in the Elderly [IQCODE]: development and cross-validation. *Psychol. Med.* 24 145–153. 10.1017/s003329170002691x 8208879

[B13] Leicht H. Berwig M. Gertz H. J. (2010). Anosognosia in Alzheimer’s disease: the role of impairment levels in assessment of insight across domains. *J. Int. Neuropsychol. Soc.* 16 463–473. 10.1017/S1355617710000056 20188013

[B14] Lewis B. D. Mills C. S. Mohs R. C. Hill J. Fillit H. (2001). Improving early recognition of alzheimer’s disease: a review of telephonic screening tools. *J. Clin. Outcomes Manage.* 8 41–45.

[B15] Lischka A. R. Mendelsohn M. Overend T. Forbes D. (2012). A systematic review of screening tools for predicting the development of dementia. *Can. J. Aging* 31 295–311. 10.1017/S0714980812000220 22877889

[B16] Lliffe S. Manthorpe J. (2004). The hazards of early recognition of dementia: a risk assessment. *Aging Ment. Health* 8 99–105. 10.1080/13607860410001649653 14982713

[B17] Luchsinger J. A. Reitz C. Honig L. S. Tang M. X. Shea S. Mayeux R. (2005). Aggregation of vascular risk factors and risk of incident Alzheimer disease. *Neurology* 65 545–551. 10.1212/01.wnl.0000172914.08967.dc 16116114 PMC1619350

[B18] Morales J. M. Gonzalez-Montalvo J. I. Bermejo F. Del Ser T. (1995). The screening of mild dementia with a shortened Spanish version of the “Informant Questionnaire on Cognitive Decline in the Elderly”. *Alzheimer Dis. Assoc. Disord.* 9 105–111. 10.1097/00002093-199509020-00008 7662322

[B19] Morales Gonzalez J. M. Gonzalez-Montalvo J. I. Del Ser Q. T. Bermejo P. F. (1992). Validation of the S- IQCODE: the Spanish version of the informant questionnaire on cognitive decline in the elderly. *Arch. Neurobiol.* 55 262–266. 1492780

[B20] Nowrangi M. A. Rao V. Lyketsos C. G. (2011). Epidemiology, assessment, and treatment of dementia. *Psychiatr. Clin. North Am.* 34 275–294. 10.1016/j.psc.2011.02.004 21536159

[B21] Petersen R. C. Smith G. E. Waring S. C. Ivnik J. Tangalos E. G. Kokmen E. (1999). Mild cognitive impairment. *Arch. Neurol.* 56 303–308. 10.1001/archneur.56.3.303 10190820

[B22] Quinn T. J. Fearon P. Noel-Storr A. H. Young C. McShane R. Stott D. J. (2021). Informant Questionnaire on Cognitive Decline in the Elderly (IQCODE) for the detection of dementia within community dwelling populations. *Cochrane Database Syst. Rev.* 7:Cd010079. 10.1002/14651858.CD010079.pub3 34278562 PMC8407460

[B23] Risacher S. L. Saykin A. J. (2013). Neuroimaging and other biomarkers for Alzheimer’s disease: the changing landscape of early detection. *Annu. Rev. Clin. Psychol.* 9 621–648. 10.1146/annurev-clinpsy-050212-185535 23297785 PMC3955298

[B24] Schulz R. Martire L. M. (2000). Family caregiving of persons with dementia: prevalence, health effects, and support strategies. *Am. J. Geriatr. Psychiatry* 12 240–249. 15126224

[B25] Stefani A. Olivola E. Bassi M. S. Pisani V. Imbriani P. Pisani A. (2013). Strength and Weaknesses of Cerebrospinal Fluid Biomarkers in Alzheimer’s Disease and Possible Detection of Overlaps with Frailty Process. *CNS Neurol. Disord. Drug Targets* 12 538–546. 10.2174/1871527311312040016 23574170

[B26] Tierney M. C. Fisher R. H. Lewis A. J. Zorzitto M. L. Snow W. G. Reid D. W. (1988). The NINCDS-ADRDA Work Group criteria for the clinical diagnosis of probable Alzheimer’s disease: a clinicopathologic study of 57 cases. *Neurology* 38 359–364. 10.1212/wnl.38.3.359 3347338

[B27] Weir D. R. Wallace R. B. Langa K. M. Plassman B. L. Wilson R. S. Bennett D. A. (2011). Reducing case ascertainment costs in U.S. population studies of Alzheimer’s disease, dementia, and cognitive impairment-Part 1. *Alzheimers Dement.* 7 94–109. 10.1016/j.jalz.2010.11.004 21255747 PMC3044596

[B28] Werner P. (2001). Correlates of family caregivers’ knowledge about Alzheimer’s disease. *Int. J. Geriatr. Psychiatry* 16 32–38. 11180483 10.1002/1099-1166(200101)16:1<32::aid-gps268>3.0.co;2-2

[B29] Wesnes K. A. Brooker H. Ballard C. McCambrigde L. Stenton R. Corbett A. (2017). Utility, reliability, sensitivity and validity of an online test system designed to monitor changes in cognitive function in clinical trials. *Int. J. Geriatr. Psychiatry* 32 e83–e92. 10.1002/gps.4659 28128869

[B30] WHO (2012). *ADI. Dementia: A Public Health Priority.* Geneve: World Health Organization.

[B31] Woods R. T. Moniz-Cook E. Iliffe S. Campion P. Vernooij-Dassen M. Zanetti O. (2003). Dementia: issues in early recognition and intervention in primary care. *J. R. Soc. Med.* 96 320–324. 10.1258/jrsm.96.7.320 12835442 PMC539533

